# Effectiveness of Case Management for Children and Young People with Mental Disorders, Substance Use and Neurodevelopmental Conditions: A Systematic Review and Multi-Level Meta-Analysis

**DOI:** 10.1192/bjo.2026.11110

**Published:** 2026-06-30

**Authors:** Wendy Shoesmith, Damilola Odipe, Naomi Thorpe, Pallab Majumder, Kapil Sayal

**Affiliations:** 1 Nottinghamshire Healthcare NHS Trust, Nottingham, United Kingdom; 2 University of Nottingham, Nottingham, United Kingdom

## Abstract

**Aims:**

To evaluate the effectiveness of case management for children and young people (CYP) with mental disorders, substance use, or neurodevelopmental conditions, and to identify components and contextual factors associated with improved outcomes.

**Methods:**

This was a systematic review and multi-level meta-analysis of randomised controlled trials (RCTs). Databases were searched from inception to June 2025. Reference lists and prior reviews were hand-searched for additional studies. Eligibility criteria: RCTs involving participants aged 5–18 years with mental health, substance use, or neurodevelopmental conditions, comparing case management (or equivalent) to treatment as usual, waiting list, or a less intensive model in community/outpatient settings. Outcomes extracted were clinical and social outcomes (which included any outcomes related to symptoms and functioning), service use (which included any outcomes related to the numberof service contacts or treatments given, excluding differences in treatments offered due to the experimental conditions), satisfaction, and cost-effectiveness. Two reviewers independently screened studies, extracted data, and assessed risk of bias using the Cochrane RoB 2 tool. Effect sizes (Hedges’ g) were pooled and meta-analyses were conducted for clinical and social outcomes and service use, applying a multilevel model to account for effect-size clustering within and between studies. Narrative review was conducted for satisfaction and cost-effectiveness. The certainty of evidence was rated using GRADE.

**Results:**

Fourteen studies were included, comprising 16 comparisons, most of which were conducted in the United States. Case management was associated with a small improvement in clinical and social outcomes (Hedges’ g=0.15; 95% CI 0.02 to 0.28) and a moderate increase in service use (g=0.68; 95% CI 0.20 to 1.17). Heterogeneity was high. Economic analyses suggested potential cost-effectiveness through reduced out-of-home placement and treatment dropout. Evidence for satisfaction was mixed. There were some concerns or high risk of bias in 10 out of 14 studies. The certainty of evidence using GRADE was very low for clinical and social outcomes and low for service use.

**Conclusion:**

The review indicates that case management can improve engagement and service use among CYP with complex mental health needs, though clinical benefits are modest, and evidence quality is limited.

